# Non-valvular infective endocarditis associated with central venous catheter bloodstream infection: a case report

**DOI:** 10.47487/apcyccv.v4i4.315

**Published:** 2023-12-27

**Authors:** Kevin Velarde-Acosta, José M. Medina-Maguiña, William Efraín Anicama Lima, Roberto Baltodano-Arellano

**Affiliations:** 1 Hospital Nacional Guillermo Almenara Irigoyen - EsSalud, Lima, Peru. Hospital Nacional Guillermo Almenara Irigoyen - EsSalud Lima Peru; 2 Facultad de Medicina, Universidad Nacional Mayor de San Marcos, Lima, Peru. Universidad Nacional Mayor de San Marcos Facultad de Medicina Universidad Nacional Mayor de San Marcos Lima Peru

**Keywords:** Endocarditis, Echocardiography, Three-Dimensional, Central Venous Catheters, Sepsis, Case Reports, Endocarditis, Ecocardiografía Tridimensional, Cateterismo Venoso Central, Sepsis, Informes de Casos

## Abstract

Non-valvular Infective endocarditis (IE) is exceedingly rare; however, its incidence has risen in tandem with the increased usage of intracardiac devices and the growing prevalence of risk factors associated with IE. We present a clinical case involving an 18-year-old patient with IE occurring at an atypical location, concomitant with central venous catheter bloodstream infection. The patient underwent targeted antibiotic therapy but ultimately required surgical resection of the vegetation due to multiple risk factors associated with a poor prognosis. This case underscores the importance of maintaining a low threshold of suspicion for IE and emphasizes the need for heightened vigilance regarding non-valvular tissues hosting foreign bodies. These less common locations pose a risk for vegetation development. Additionally, we underscore the pivotal role of 3D echocardiography tools in anatomically characterizing the vegetation, including dimensions, implantation area, and related anatomy. These tools provide realistic images that facilitate informed decision-making. Furthermore, the timely selection of surgical intervention in patients at elevated risk of therapeutic failure is a cornerstone in effective management.

## RESUMEN

La endocarditis infecciosa (EI) no valvular es extremadamente infrecuente; sin embargo, su incidencia ha aumentado paralelamente al incremento del uso de dispositivos intracardiacos y a la creciente prevalencia de factores de riesgo asociados a la EI. Se presenta un caso clínico de un paciente de 18 años con EI en una localización atípica, concomitante con infección del torrente sanguíneo por catéter venoso central. El paciente fue sometido a un tratamiento antibiótico específico, pero finalmente requirió la resección quirúrgica de la vegetación debido a múltiples factores de riesgo asociados a un mal pronóstico. Este caso resalta la importancia de mantener un umbral bajo de sospecha de EI y hace hincapié en la necesidad de una mayor vigilancia de los tejidos no valvulares que albergan cuerpos extraños. Estas localizaciones menos frecuentes suponen un riesgo para el desarrollo de vegetación. Asimismo, subrayamos el papel fundamental de las herramientas de ecocardiografía tridimensional en la caracterización anatómica de la vegetación, incluidas las dimensiones, el área de implantación y la anatomía relacionada. Estas herramientas proporcionan imágenes realistas que facilitan la toma de decisiones informadas. Además, la selección oportuna de la intervención quirúrgica en pacientes con alto riesgo de fracaso terapéutico es una piedra angular en el manejo eficaz.

## Introduction

Infective endocarditis (IE) is a significant public health problem, estimated to affect 3 to 10 individuals per 100,000 population annually, with high hospital costs per patient ^1^. IE is also a potentially fatal disease, contributing to approximately 66,300 deaths worldwide ^1^. Given its high morbidity and mortality, the identification of different risk factors and preventive strategies is crucial for the timely detection of this disease.

In recent years, the population at risk has increased, leading to new clinical scenarios in the presentation of IE ^2^^-^^4^. The rise in interventional procedures and the utilization of intracardiac devices have given rise to new locations and foci as potential starting points for the development of IE ^(^^5^.

In this context, we present a case of non-valvular infective endocarditis, emphasizing several risk factors contributing to its development, the atypical location of the infectious focus, and the significance of maintaining a low threshold of suspicion. Additionally, we highlight the usefulness of echocardiographic tools for the proper identification and characterization of vegetation.

## Case report

An 18-year-old male patient was admitted to the emergency room due to fever and arterial hypotension during hemodialysis. His medical history included end-stage renal disease (ESRD) on hemodialysis due to congenital bilateral hydronephrosis and recurrent episodes of bacteremia associated with a central venous catheter (CVC-BSI).

On physical examination, he exhibited tachycardia, tachypnea, warm, earthy skin, bilateral subconjunctival hemorrhage, rhythmic heart sounds, and no aggregate sounds. Laboratory tests revealed leukocytosis, left shift, and elevated inflammatory markers. Blood cultures confirmed bacteremia due to methicillin-resistant *Staphylococcus Aureus* (MRSA).

**Figure 1:**
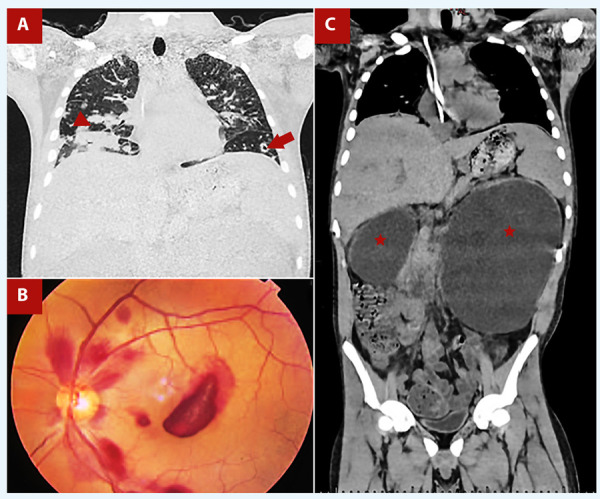
A. Coronal chest tomography shows a right lower lobe pneumonic consolidation (red arrowhead) and a peripheral cavitated nodule suggestive of a septic emboli (red arrow). B. Fundus photography showing a cotton wool spot surrounded by hemorrhage (Roth spot), due to septic emboli at the retina vessels. C. Thoraco-abdominal tomography shows severe bilateral hydronephrosis predominantly on the left (red stars). Likewise, a central venous catheter is evident.

The electrocardiogram revealed sinus tachycardia without other significant signs. Thoracic CT showed multiple nodules, some cavitated, in both lung fields, predominantly peripheral, suggestive of pulmonary septic emboli and a pneumonic consolidation in the right lower lobe **(**Figure 1A**)**; while the abdominal CT determined severe bilateral hydronephrosis and distortion of the renal parenchyma **(**Figure 1C**)**. Fundus photography was performed looking for septic emboli at the retina (Roth spots) **(**Figure 1B**)**. 

**Figure 2:**
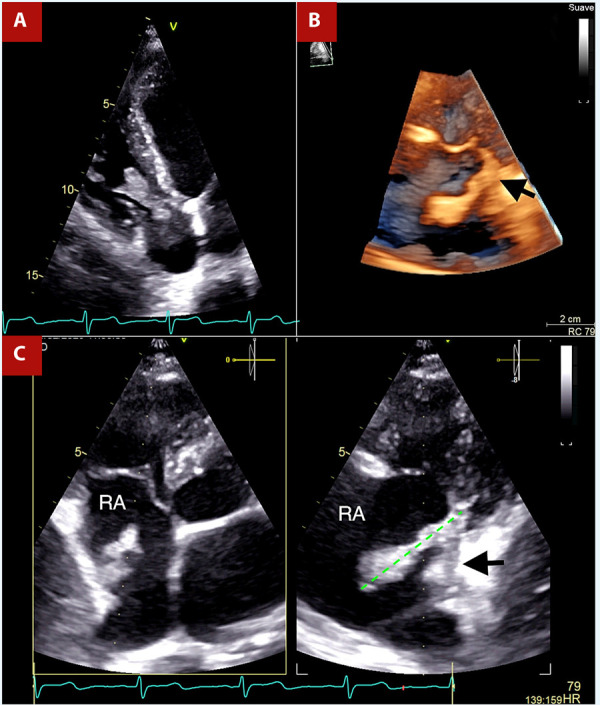
A. TTE shows a finger-like mass in the right cavities. B. Volume rendering of the mass implanted near the tricuspid annulus (black arrow). C. X-plane focused on the right atrium, orthogonal image evidence inflammatory tissue in the wall of the right atrium from which a vegetation emerges (black arrow), Likewise, it is useful to measure the vegetation, 35 mm (green line).

Given the high suspicion of infective endocarditis, transthoracic echocardiography (TTE) was performed, revealing a 35mm long, digitiform vegetation emerging from the posterior wall of the right atrium, just above the tricuspid annulus, that protrudes into the right ventricle in diastole without functionally affecting the tricuspid valve **(**Figure 2A**,** Video 1**)**. The 3D-volume rendering provided a realistic representation of the vegetation’s proximity to the tricuspid ring **(**Figure 2B**,** Video 2**)**. The X-plane tool, in the orthogonal image, showed inflammatory tissue covering the lower half of the posterior wall of the right atrium, emerging towards the cavity as a vegetation (Video 3**)**. In addition, it allowed us to confirm its maximum length **(**Figure 2C**)**. Otherwise, it was a study without additional relevant findings.

With two major and three minor criteria for the modified Duke score, a diagnosis of non-valvular infective endocarditis was established. New blood cultures were taken at different times of the day, and empirical antibiotic treatment was started with cefepime (1g IV qd) and vancomycin (loading dose 20 mg/kg IV once, maintenance dose 750 mg IV after each dialysis). Subsequently, upon repeatedly isolation of methicillin-resistant *Staphylococcus Aureus* in > 3 blood cultures, treatment with vancomycin alone was maintained for approximately one week. However, the team decided on surgical resection of the vegetation and inflammatory tissue due to factors associated with poor prognosis. 

**Figure 3:**
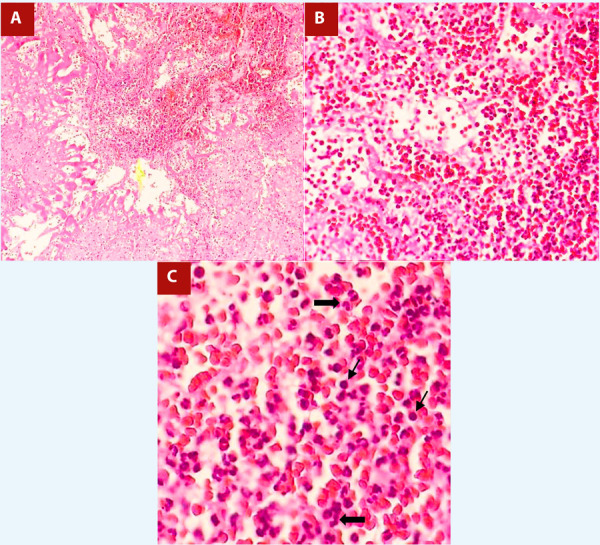
A. Blood clot with presence of lymphoplasmacytic infiltrate. HE 10x. B, C. At higher magnification, polymorphonuclear proliferation (thick arrows) and isolated plasmatic cells (thin arrows) are seen, as well as a large number of red blood cells. HE 40x and 80x.

During surgery, only remnants of the vegetation were found on the wall of the right atrium, adjacent to the tricuspid septal leaflet (without compromising it) and at the inlet of the inferior vena cava, which corroborated the presurgical echocardiographic findings. Due to the previous events of septic embolism, we hypothesize the migration of the vegetation prior to or during surgery or inadvertent aspiration of the vegetation during entry into the right atrium. The histological study of the surgical specimen showed a blood clot with an acute inflammatory infiltrate, consistent with acute bacterial endocarditis **(**Figure 3A**,**3B**,**3C**)**. Likewise, the vegetation was sent to culture without obtaining microbiological results, probably due to the antibiotic scheme established. The patient had a favorable postoperative evolution and completed antibiotic treatment for 3 more weeks. No adverse and unanticipated events occurred.

**Figure 4:**
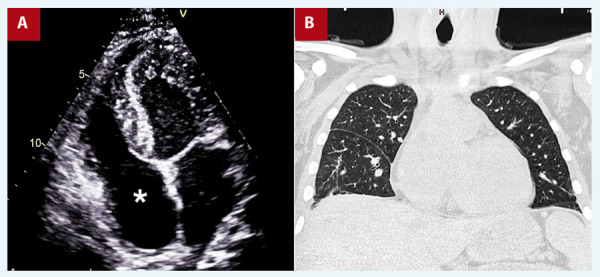
A. TTE, 4-chamber. No vegetation is visible in the right atrium (asterisk). B. Chest tomography without evidence of inflammatory lesions. Ventral venous catheter not visualized.

At 6-month follow-up, the patient reported no cardiac symptoms, the echocardiography showed no vegetations **(**Figure 4A**,** Video 4**)**, and chest tomography showed no parenchymal lesions **(**Figure 4B**)**.

## Discussion

IE is a potentially fatal disease that typically affects the valvular endothelial tissue of the heart. Right-sided IE represents only 5% to 10% of all IE cases, and there is almost always valvular involvement ^(^^6^. Several risk factors associated with right-IE have been described, including parenteral drug addiction, patients with HIV seropositivity or immunosuppressed patients, and the presence of intracardiac devices (pacemakers, intra-cardiac defibrillators, vascular access for hemodialysis) ^(^^6^. The latter has emerged as a higher risk factor for infection and bacteremia, with the highest incidence with temporary CVCs and the lowest among permanent native arteriovenous fistulas or synthetic grafts ^(^^7^. Patients with ESRD on HD also present an increased risk of IE due not only to the presence of vascular accesses and immunosuppression but also to degenerative changes in the heart valves ^(^^8^.

Even despite the presence of risk factors for right-IE, the vast majority of cases are in the left heart chambers. In a study by Hoen *et al.* ^(^^9^ on the prevalence of IE locations, the right atrium wall is not even listed, probably because of the rarity of the finding. However, some later reports ^(^^8^^,^^10^^,^^11^^)^ describe similar cases to this one presented, including even cavo-atrial involvement.

It is imperative to keep in mind that right atrial masses are rare entities with a broad differential diagnosis, including tumors, vegetation, and thrombus ^(^^12^. However, due to the risk factors present (ESRD on HD and history of multiple CVC-BSI), the clinical picture, and the positive modified Duke criteria in this case, a presumptive diagnosis of non-valvular IE was made.

In our patient, conservative management with targeted antibiotic treatment was initially planned. However, given the presence of factors associated with high morbidity and mortality (MRSA infection, pulmonary metastatic infection, persistence of fever despite treatment with vancomycin and vegetation > 20 mm) and the absence of tricuspid valve involvement, surgical management was chosen, with a very favorable outcome.

We report the case, in the first place to show an atypical and rare location of implantation of the IE vegetation, such as the right atrial wall. Secondly, emphasize the role of cardiovascular devices as emerging and relevant risk factors for infective endocarditis, particularly of the right cavities. Finally, to highlight the usefulness of 3D tools, such as the X-plane, to determine anatomical details such as the location of the vegetation’s implantation zone and the rendering volume that provides realism and illustrates the related anatomy, all of which are useful for surgical planning.

In conclusion, although extraordinarily rare, non-valvular IE can occur in patients with ESRD on hemodialysis through a central line. Vigilance is crucial for nonvalvular cardiac tissues in contact with cardiovascular devices, where vegetations can potentially form. 3D echocardiography tools play a fundamental role in anatomically characterizing vegetations and aiding surgical planning (dimensions, implantation area, related anatomy, and realistic images). In cases with a high probability of therapeutic failure and morbimortality, surgical treatment should be considered.
